# Phage‐Inducible Chromosomal Islands as a Diagnostic Platform to Capture and Detect Bacterial Pathogens

**DOI:** 10.1002/advs.202301643

**Published:** 2023-06-26

**Authors:** Rodrigo Ibarra‐Chávez, Julien Reboud, José R. Penadés, Jonathan M. Cooper

**Affiliations:** ^1^ Department of Biology Section of Microbiology University of Copenhagen Universitetsparken 15, bldg. 1 Copenhagen DK2100 Denmark; ^2^ Institute of Infection Immunity and Inflammation College of Medical Veterinary and Life Sciences University of Glasgow Glasgow G12 8TA UK; ^3^ Division of Biomedical Engineering James Watt School of Engineering University of Glasgow Glasgow G12 8QQ UK; ^4^ Departamento de Ciencias Biomédicas Universidad CEU Cardenal Herrera Moncada 46113 Spain; ^5^ Centre for Bacterial Resistance Biology Imperial College London South Kensington SW7 2AZ UK

**Keywords:** bacterial detection, diagnostics, mobile genetic elements, paper microfluidics, phage satellites, PICIs

## Abstract

Phage‐inducible chromosomal islands (PICIs) are a family of phage satellites that hijack phage components to facilitate their mobility and spread. Recently, these genetic constructs are repurposed as antibacterial drones, enabling a new toolbox for unorthodox applications in biotechnology. To illustrate a new suite of functions, the authors have developed a user‐friendly diagnostic system, based upon PICI transduction to selectively enrich bacteria, allowing the detection and sequential recovery of *Escherichia coli* and *Staphylococcus aureus*. The system enables high transfer rates and sensitivities in comparison with phages, with detection down to ≈50 CFU mL^−1^. In contrast to conventional detection strategies, which often rely on nucleic acid molecular assays, and cannot differentiate between dead and live organisms, this approach enables visual sensing of viable pathogens only, through the expression of a reporter gene encoded in the PICI. The approach extends diagnostic sensing mechanisms beyond cell‐free synthetic biology strategies, enabling new synthetic biology/biosensing toolkits.

## Graphical abstract

In this report, a new PICI‐based platform is showcased to detect and identify bacteria. In contrast to other diagnostic approaches, this strategy can additionally distinguish whether the pathogens are alive. PICI‐based infection of target hosts is simple and efficient, allowing us to demonstrate the implementation of the technique in an easy‐to‐use paper‐based format.

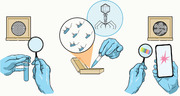

## 1 Introduction

Bacterial infectious diseases have become a major global health concern with antimicrobial resistance causing an upsurge in their prevalence and severity.^[^ ^1^ ^]^ New strategies are now required not only for the detection and identification of bacterial pathogens in humans and animals but also for a better understanding of their mobility in the environment through microbial ecosystems.^[^ ^1^, ^2^ ^]^ While detection can be accomplished through resource‐intensive methods such as membrane filtration, tube fermentation, and biochemical assays (including those involving molecular tests),^[^ ^3^ ^]^ there is an urgent need to develop affordable and rapid diagnostics, which can be used in low‐resource settings where fixed infrastructure and centralized laboratories are lacking.^[^ ^4^ ^]^

The impact of bacterial pathogens is not just evident in human and animal health. Recent upsurges in bacterial foodborne outbreaks now affect the supply chain of daily life products, challenging food security whilst bringing significant economic loss (with the cost of incidents reported to be valued at ≈$7 billion and with an annual estimate of hundreds of thousands of hospitalized consumers).^[^ ^5^ ^]^

Applications for low‐cost point‐of‐care or point‐of‐need diagnostics have previously been developed using low‐cost paper microfluidics,^[^ ^6^, ^7^ ^]^ including those to identify bacteria using phages.^[^ ^8^, ^9^, ^10^, ^11^ ^]^ Formerly, the strategy of using phages in diagnostics has arisen through a process of co‐evolution where the virus has adapted to the complex lifestyles of its bacterial host.^[^ ^12^, ^13^, ^14^ ^]^ This close relationship generally affords a key analytical advantage in the specific detection of the bacteria,^[^ ^15^ ^]^ particularly when the phage has been genetically engineered to carry reporter genes for recognition,^[^ ^16^ ^]^ although these strategies necessarily have a limited host range. Notwithstanding these limitations, phage‐based strategies have been incorporated into paper‐based devices as biosensors to visualize and quantify the presence of pathogens in a variety of samples with the key advantages being their low cost as well as the disposable and portable nature of the technology.^[^ ^8^, ^9^, ^17^, ^18^ ^]^

PICIs are a widespread family of mobile genetic elements (MGEs), which have an important role in bacterial pathogenesis and phage interference,^[^ ^19^, ^20^ ^]^ hijacking their phage capsids and spreading amongst other bacterial communities at extremely high frequencies. Recently, it has been shown that PICIs are present in more than 200 different bacterial species,^[^ ^21^ ^]^ making them a highly attractive and adaptable vehicle that could be used for the detection of many different pathogens.

In this report, we showcase a novel application of the PICIs which, in contrast to phages, offer the advantage of infecting and integrating with its host during both exponential and stationary growth stages and without lysing the organism (as phages do).^[^ ^22^ ^]^ By showing that these elements can be maintained in their host and promote sufficient expression of a reporter gene, we demonstrate that we can achieve viable bacterial detection, while avoiding the spread of undesirable traits, such as antibiotic‐resistant genes. To illustrate this potential, we focus on the demonstration of the detection of *Escherichia coli (E. coli)* and *Staphylococcus aureus (S. aureus)* as model bacteria.

Conventional methods for bacterial detection using nucleic acid‐based molecular amplification methods currently report both live and dead bacteria indiscriminately (with nucleic acid biomarkers from previously lysed microorganisms being detected alongside viable bacteria). Although “viability‐PCR” approaches have previously been described to only detect live bacteria, illustrating the importance of being able to determine the viability of organisms, these strategies are complex to implement and require fixed laboratory infrastructure.^[^ ^23^ ^]^ This proposed new PICI‐based platform contrasts with existing cell‐free synthetic biology strategies,^[^ ^24^ ^]^ using the pathogen directly as a signal transducer, and allowing the identification of only live pathogens.

By developing a paper microfluidic assay format, we also demonstrate the potential for this application in low‐resource settings with decentralized infrastructure, as might be needed for food safety and environmental monitoring, as an indicator of inadequate hygiene and the potential risk of contaminated food and water.^[^ ^25^ ^]^ We propose that PICIs are unexplored elements suitable for both point‐of‐care/point‐of‐need and therapeutic applications without the requirements for cold‐chain logistics, associated with other strategies.

## 2 Results

### 2.1 Limit of Detection by PICIs

PICIs are found in many different species with the most well‐characterized being found in *E. coli* and *S. aureus*.^[^ ^26^ ^]^ The Staphylococcal PICIs, also known as Staphylococcal pathogenicity islands (SaPIs), are the prototypical family of PICIs.^[^ ^20^ ^]^ Their well‐studied gene architecture led to the discovery of different classes in other bacterial species with different strategies to parasitize and hijack their helper phage. Such is also the case of the PICIs found in *E. coli* and related species, denominated EcCIs,^[^ ^26^ ^]^ some of which can hijack coliphages like phage *λ*, a widely used phage to engineer particles for detection and gene delivery.^[^ ^27^, ^28^, ^29^, ^30^, ^31^ ^]^ Recently, both PICI types have been used as a potential antimicrobial alternative by replacing the pathogenicity genes with CRISPR‐Cas genes.^[^ ^32^, ^33^ ^]^ In view of this, we sought to demonstrate their use for the detection of such pathogens, as a new bioanalytical platform.

As an initial step, we investigated the limit of detection (LOD) that PICIs can achieve to discriminate between the presence or absence of a pathogen, by transferring their DNA into a recipient bacterium. To measure the transfer and integration of the PICI DNA in the recipient cells, we generated several PICIs carrying different antibiotic‐resistant markers, to facilitate the transfer studies (See Table S2 (Supporting Information) for transduction rates). Specifically, we introduced these markers in SaPIbov1 and EcCICFT073, creating SaPIbov1 *sec*::*erm*C and EcCICFT073 *c1504‐c1507::cat*, respectively. The PICI infective particles were produced using mitomycin C to induce the cognate helper phages and a package of PICI DNA in the phage capsids from the donor strains. These donor strains can only produce PICI particles since the cognate helper phages contain a mutation that impairs their ability to pack their own phage DNA.^[^ ^34^, ^35^, ^36^ ^]^ The rationale behind using a packaging defective helper phage was to avoid the interference created by phages, which can potentially trigger lysis of the recipient cells, reducing the sensitivity of these assays. The PICI particles were released out of the cells, and the lysates were filtered to enable the quantification of transfer units per mL of the lysate (or transduced forming units TFU mL^−1^) using a recipient strain (RN4220 for *S. aureus* and 594 for *E. coli*), normalised to a transfer unit of ≈10^6^ TFU mL^−1^.

**Figure 1:**
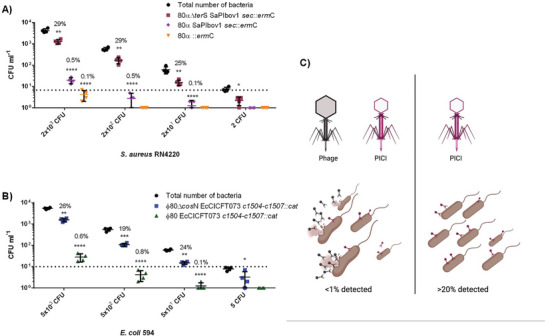
Limit of detection using PICIs. Graphs reveal the overall performance of using PICIs SaPIbov1 *sec*::*erm*C and EcCICFT073 *c1504‐c1507::cat*. The number of transduced cells obtained from each lysate was compared to the total number of cells (black) to obtain the percentage of detected bacteria used in the assay. All PICI, phage/PICI and transducible phage lysates were normalized at ≈10^6^ TFU mL^−1^. A) Concentration of *S. aureus* RN4220 was used within the range of 2 × 10^3^–2 CFU mL^−1^ to measure the limit of detection of a PICI‐only (80*α* Δ*ter*S SaPIbov1), phage‐PICI lysate (80*α* SaPIbov1) and phage lysate (80*α*::*erm*C). Statistical analysis was performed using one‐way ANOVA followed by Tukey's multiple comparison tests (*n* = 4 ± SD, **p = 0.0043 for 2 × 10^3^ CFU, **p = 0.0014 for 2 × 10^2^ CFU; **p = 0.0019 for 2 × 10^1^ CFU, *p = 0.0230 for 2 CFU, ****p < 0.0001). Error bars represent the standard deviation of the mean. B) Concentration of *E. coli* 594 was used within the range of 5 × 10^3^–5 CFU mL^1^ to measure the limit of detection of a PICI‐only (80 Δ*cos*N EcCICFT073) and phage‐PICI lysate (*ϕ*80 EcCICFT073). Statistical analysis was performed using one‐way ANOVA followed by Tukey's multiple comparison tests (n = 4 ± SD, **p = 0.0011 for 5 × 10^3^ CFU, ***p = 0.0002 for 5 × 10^2^ CFU; **p = 0.0039 for 5 × 10^1^ CFU, *p = 0.0220 for 5 CFU, ****p < 0.0001). Error bars represent the standard deviation of the mean. C) Employing phage particles containing phage DNA or lysates with active temperate phages and PICI DNA, cultures get severely affected by the stochastic effect of lysis activation which decreases the viability of cells and the limit of detection (<1%). Phage particles containing only PICI DNA enable higher detection (>20%) percentage and do not affect the viability of the cells.

To track the transfer of the PICI DNA with different concentrations of cells, we established cultures with predetermined numbers of cells and then exposed these to infection of a PICI lysate, using a standard dilution. The percentage of transduced bacterial cells (TFU mL^−1^) relative to the total number of cells (CFU mL^−1^) was determined, in **Figure** **1**. For the detection of *S. aureus*, we were able to obtain transfer as low as ≈2 CFU mL^−1^, however, the variability for detection was higher than ≈20 CFU mL^−1^, which was determined as the LOD for SaPIbov1. On the other hand, using EcCICFT073, we were able to detect as low as ≈5 CFU mL^−1^ of *E. coli*. The percentages of transduced cells compared to the total of predetermined cells ranged between 19–29%, suggesting that a quarter of the total cells had been infected and the PICI was successful to integrate and express the antibiotic resistance gene.

We optimized the multiplicity of infection (MOI) in the assay to establish the number of PICI particles transduced and detected relative to the number of total cells (≈200 or ≈500 cells used in the assay). Importantly, we achieved the detection of transduced cells at lower dosages of PICI‐lysate, suggesting that even at lower MOIs (10:1 and 1:1), we were able to detect 21 and 3 viable cells respectively (Figure S1, Supporting Information). Typically, a culture of donor cells can produce ≈10^8^–10^6^ TFU mL^−1^, which is sufficient to add into the detection system without worrying that detection levels could reduce if PICI particles are destroyed while distributing or using the system (Figure S1, Supporting Information). We also tested lysates using different helper phages to transfer our PICIs, as some of these viral particles employ different abilities to mobilize the PICI DNA. The highest percentage achieved of detected *S. aureus* cells was 39.4% using *ϕ*NM1 *ΔterS* SaPIbov1 and 33.8% using 80*α ΔterS* SaPIbov1 and for *E. coli* was 22.2% using *ϕ*80 *ΔcosN* EcCICFT073 (Figure S2, Supporting Information). For *E. coli* this may be slightly lower since transfer rates of the EcCICFT073 island are 10^7^ TFU mL^−1^, as the inability of the helper phage (Δ*cos*N) to be packed did not improve EcCI packaging considerably, as it does in SaPIs with Δ*ter*S phages achieving >10^8^ TFU mL^−1^.^[^ ^34^, ^35^, ^36^ ^]^ Nonetheless, this mutation impeding the *E. coli* prophage packaging allowed higher sensitivity (from 3–7 to 70–140 CFU mL^−1^) given more viable cells were available as the helper phage could lyse them (Figure 1; Figure S2, Supporting Information).

To compare the detection by transduction of PICI DNA versus a temperate phage, which will integrate with the recipient cells,^[^ ^37^, ^38^ ^]^ we used a marked Siphovirus (80*α*::*erm*C) to induce SaPIs. Detection of *S. aureus* by transfer of phage DNA was only seen in samples with at least ≈2 × 10^3^ CFU mL^−1^ with a detection rate of 0.1%, meaning that less than 10 TFU mL^−1^ can retain the phage and grow from an initial number of ≈2 × 10^3^ CFU mL^−1^, Figure 1A. These experiments indicate that PICIs were able to detect ≈100‐fold more viable bacteria than the helper phage, Figure 1 and Figure S2 (Supporting Information). We hypothesize that this was a consequence of the ability of the phage to lyse many of the recipient cells before integrating the bacterial chromosome. Indeed, when the PICI DNA transfer was analyzed in the presence of the helper phage (and not using a defective phage incapable of being packaged), the efficacy of the PICIs was severely reduced. In the presence of the inducing phage, PICI transfer was <20%, Figure S2 (Supporting Information), and in some combinations dropping to ≈0.5%, Figure 1, when compared to the total amount of *S. aureus* cells. For *E. coli* cells, transductions were <2% when infecting ≈500 CFU mL^−1^ (Figure 1; Figure S2, Supporting Information). Here, the difference observed in transductions with the same PICI employing different inducing helper phages is due to the stochastic lytic induction that these have.^[^ ^39^, ^40^, ^41^ ^]^

### 2.2 PICI Transduction on Microfluidic Paper‐Based Analytical Devices (*µ*PADs)

Paper‐based devices have proven to be an important tool during the COVID‐19 pandemic, coupled with RT‐PCR, and enabling use in communities and remote locations or resource‐poor areas, while providing a rapid, portable, and easy‐to‐handle in field‐based diagnostic.^[^ ^42^, ^43^, ^44^ ^]^ Similarly, diseases such as malaria in humans^[^ ^45^, ^46^ ^]^ and bovine herpes viruses (BoHV‐1) in cattle^[^ ^44^ ^]^ have been detected using similar technologies. We, therefore, aimed to adapt the transduction of PICIs for the detection of bacteria into a paper‐based device that could serve as a stable platform for their distribution and easy readout.

**Figure 2:**
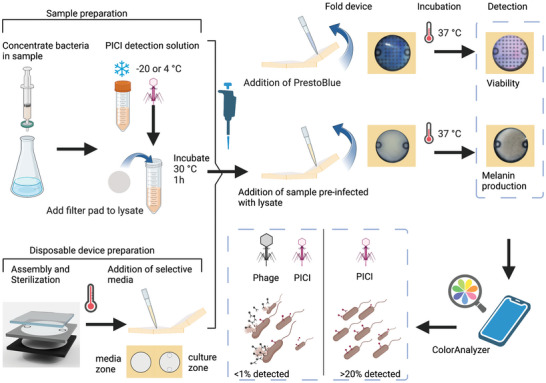
Set up of bacteria detection by PICIs on a portable culture device. Preparation of detection solution with PICIs: PICI lysates are generated by growing bacteria and inducing the SOS response by adding mitomycin C. The PICI lysate can be stored until needed at 4 or −20 °C. Disposable device preparation: devices are fabricated accordingly and sterilized containing dH_2_O, these are then opened on a sterile zone and addition of selective. PrestoBlue can be added to allow detection by viability, and disposable *µ*PADs can be stored and shipped for bacterial detection. Sample preparation: Low numbers of bacteria in a liquid sample can be concentrated onto a filter pad. Once bacteria are retained, the filter pad can be submerged into a detection solution with concentrated PICI to allow infection. The detection solution is vortexed with the filter pad, incubated at 30 °C or room temperature for 1 h, and 200–500 µL are added to the culture zone of the portable culture device and folded to allow contact between the culture zone and the media zone. Portable devices are incubated at 37 °C overnight and analyzed according to the synthetic circuit used for the detection (melanin production or viability by antibiotic selection). Bacterial quantification is performed by taking images from portable devices using a smartphone camera and processed with ColorAnalyzer Mobile App. The figure was created with Biorender.com.

We adapted a previous design targeted at bacteria culture,^[^ ^8^, ^18^ ^]^ and compared different conditions for culture and validity of PICI DNA transduction. The paper‐based microfluidic devices used in our set‐up work as both Petri dishes and the diagnostic sensor; where the combination of paper sheets with hydrophobic wax barriers, low‐cost tape, and silicone polymer membrane made of polydimethylsiloxane (PDMS) to facilitate moisture retention and oxygen diffusion, while allowing growth by having nutritious media permeated into the paper sheets. The PDMS membrane functions as a barrier to avoid contamination and as a transparent optical window to allow an easy readout for the users, Figure S3 (Supporting Information). See **Figure** **2** for the procedure on how to set up portable devices for detection with PICIs.

To compare transduction on‐chip with the classic transduction on a plate, we quantified the transfer of two different lysates for SaPIbov1 *tst*::*tet*M and two different lysates for EcCICFT073 *c1504‐c1507::cat*. Briefly, we used 1 mL of the recipient cells and 100 µL of PICI lysate, incubated for 5 h at 37 °C and proceed to serial dilute and plated on agar containing selective media supplemented with antibiotics; 3 µg mL^−1^ tetracycline for SaPIbov1 *tst*::*tet*M transfer and 20 µg mL^−1^ chloramphenicol for EcCICFT073 *c1504‐c1507::cat* transfer (see PICI transduction in Experimental Section for more details).

For transduction on‐chip, we tested two different procedures, namely: first, performing an incubation in liquid of 1 mL of recipient cells and PICIs together for 20 min followed by transferring 100 µL to the culture zone on the *µ*PADs (as depicted in Figure 2); or alternatively using 100 µL of recipient bacteria followed by applying 100 µL of PICI lysate directly into the culture area. Portable devices were sealed and incubated at 37 °C for 5 h, and after incubation, the devices were opened in front of a Bunsen burner, on top of a sterile surface, and the culture and media areas were cut from the device using hot tweezers. Both areas were put into a tube with 1 mL of media and vortexed for 5 min. Following the release of cells into the liquid, we made serial dilutions and plated using appropriate top agar on TSA or LB agar plates with selective antibiotics for each technical replicate. All plates were incubated at 37 °C and, after 12 h, colonies were counted and the number of TFU mL^−1^ was estimated.

**Figure 3:**
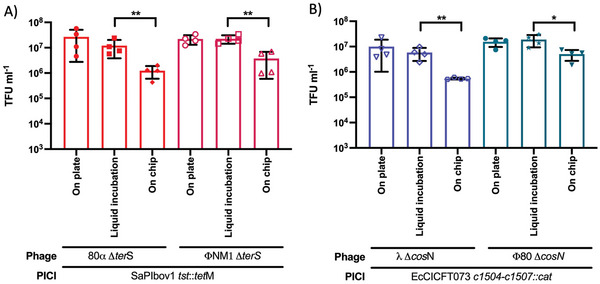
PICI infection on‐a‐chip has similar transduction titers to traditional assays. A) Detection of *S. aureus* strain RN4220 transduced with 80*α ΔterS* SaPIbov1 *tst::tet*M (red) or *ϕ*NM1 *ΔterS* SaPIbov1 *tst::tet*M (magenta). Statistical analysis was performed using one‐way ANOVA followed by Tukey's multiple comparison tests (*n* = 4 ± SD, **p = 0.0016 for comparison of 80*α ΔterS* SaPIbov1, **p = 0.0035 for comparison of *ϕ*NM1 *ΔterS* SaPIbov1). B) Detection of *E. coli* strain 594 transduced with *λ Δcos*N EcCICFT073 *c1504‐c1507*::*cat* (blue) or *ϕ*80 *Δcos*N EcCICFT073 *c1504‐c1507*::*cat* (turquoise). Statistical analysis was performed using one‐way ANOVA followed by Tukey's multiple comparison tests (*n* = 4 ± SD, **p = 0.0038 for comparison of *λ Δcos*N EcCICFT073, *p = 0.0345 for comparison of *ϕ*80 *Δcos*N EcCICFT073). Error bars represent the standard deviation of the mean.

When comparing our two procedures, we confirmed that PICIs can infect and transduce on the paper microfluidic devices, **Figure** **3**, as we did not observe a significant difference between transductions performed on plates or liquid incubation before adding samples on µPADs for 5 h incubation (≈10^7^ TFU mL^−1^ for SaPIbov1 and EcCICFT073 respectively in both instances), while transduction on devices with non‐incubated samples and direct addition of lysate in µPADs exhibited a tenfold reduction than samples incubated for 20 min with 100 µl of the lysate (e.g., *λ ΔcosN* EcCICFT073 *c1504‐c1507::cat* transfer reduced from ≈10^6^ to 3 × 10^5^ TFU mL^−1^).

Direct addition of PICI particles into the paper device is the best implementation of the strategy as it minimizes user input. However, it resulted in decreased efficiencies potentially due to obstruction, impeding PICIs from infecting. Nevertheless, direct addition enabled sufficient performance and crucially will allow for easier implementation. The devices can be stored outside the cold chain, as we have observed that PICIs are stable for few months at room temperature and that these can be stored at 4 and −20 °C without having evident effects on PICI titer, Figure S4 (Supporting Information).

### 2.3 Calibration of On‐Chip Detection using PrestoBlue

Having proven that PICIs can transduce to their recipient host in the paper microfluidic devices, we performed both infection and estimation of transduced cells, using a cell viability reagent to allow us to quantify the cells that were able to grow on the device with antibiotics after been transduced with PICIs and acquired the antibiotic resistance maker. PrestoBlue is a fast resazurin‐based stain used as a growth indicator for prokaryotic cells, enabling a clear distinction between viable cells and nonviable cells. This reagent is commonly used to monitor living cells in toxicity assays,^[^ ^47^ ^]^ and enabled us to determine the LOD by the viability of the transduction‐on‐chip and provide an easy visual readout.

**Figure 4:**
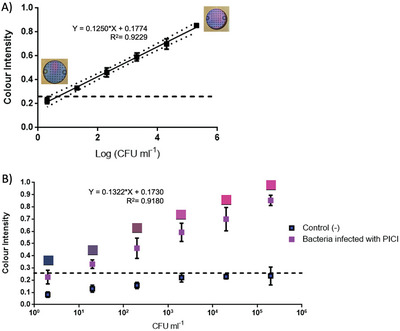
Calibration curves for detection of bacteria on paper microfluidic devices. Samples containing bacteria cells were loaded on devices containing media (LB or TSB) and antibiotics when needed. A) Calibration curve for the detection of bacteria using PrestoBlue. Known concentrations of either *S. aureus* or *E. coli* were added to the disposable devices and cultured overnight at 37 °C followed by the development of colour by adding PrestoBlue and using ColorAnalyzer on an iPhone 5s camera to obtain L (lightness), a* (green‐red scale) and b* (yellow‐blue scale) were recorded to then calculate the relative sRGB (standard Red Green Blue) value. The results are the mean of *n* = 5 ± SD with a dotted line representing a 95% confidence interval. B) Transduction on‐chip of PICI with a defined quantity of bacteria. Several samples with known concentrations of bacteria were incubated with PICIs at a high MOI previously added onto *µ*PADs. Samples with a mix of bacteria and PICI on‐chip were incubated overnight at 37 °C. Transduced cells were able to grow on *µ*PADs with antibiotics and were detected with PrestoBlue. Colour squares are the representation of the sRGB values obtained. Negative controls were samples of recipient cells without PICI infection. Error bars represent the standard deviation of the mean.

To detect the color pattern reflecting the related concentration of bacteria, PrestoBlue was added to culture zones and portable devices were incubated for 2 h to develop the color. Images of the portable device were acquired using ColorAnalyzer Mobile App and EasyRGB to generate a calibration curve by relating the color intensity with the exponential quantity of bacteria added, **Figure** **4**A. Here the LOD was determined as 0.260 (representing the mean plus three standard deviations above the average control background, that is, 0.125 + 3 × 0.045).

Having determined the LOD on the paper microfluidic devices with PrestoBlue, we used PICIs with antibiotic resistance cassettes, namely SaPIbov1 *tst*::*tet*M and EcCICFT073 *c1504‐c1507::cat*, to only permit growth and detection of the bacteria that had been transduced with a PICI. Samples with a known concentration of recipient strains from the range of 10^1^–10^6^ were incubated with a lysate of SaPIbov1 (10^7^ TFU mL^−1^) or EcCICFT073 (10^6^ TFU mL^−1^) for 20 min and added to the portable devices with TSB with 3 µg mL^−1^ tetracycline for *S. aureus*, and LB with 20 µg mL^−1^ chloramphenicol. The portable devices were incubated overnight at 37 °C to then be developed with PrestoBlue. The cells infected and transduced with the corresponding PICI gained the antibiotic resistance cassette, which allowed them to exponentially grow on the portable device and develop the color change from blue to magenta. We used samples of the same quantity of bacteria without the addition of PICI as negative controls, illustrated in Figure 4B as blue squares below the LOD dotted line.

Similar to the calibration curve for growth, Figure 4A, transduction on‐chip followed an adequate pattern where the intensity of the color reflects the number of bacteria present in the sample, Figure 4B. Here, bacterial growth was enabled by the transfer of the PICI and expression of the resistance gene to the antibiotic supplemented in the paper microfluidic devices. The color values obtained from EasyRGB were represented as squares on top of each plot point, illustrating the color development for each concentration of bacteria, Figure 4B. The LOD was ≈50 CFU mL^−1^, which was higher than in previous experiments performed on plates (≈5 CFU mL^−1^), perhaps representing a shortcoming of the detection mechanism in the paper microfluidic device (as the two calibration curves suggest that the sensitivity, as the gradient of the linear regression, for both cultured bacteria on‐chip and samples used to detect bacteria with PICI infection on‐chip are similar).

### 2.4 Sensitivity of PICI‐Based in Paper Microfluidic Devices

**Figure 5:**
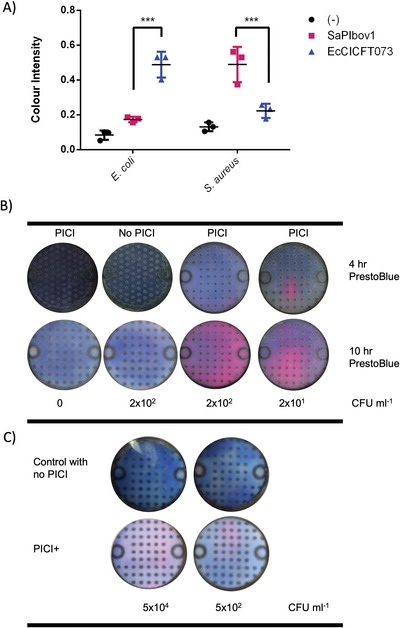
Detection of bacteria enabled by transduction of PICIs and PrestoBlue in *µ*PADs. Samples containing bacteria cells were loaded on devices containing media (LB or TSB) and antibiotics, A) Ability to detect between two bacterial species in mixed bacterial cultures was tested by adding *E. coli* 598 (10^2^ CFU mL^−1^) and *S. aureus* RN4220 (10^2^ CFU mL^−1^) to *µ*PADs with PICIs SaPIbov1 or EcCICFT073. Detection was enabled by the specific infection of *S. aureus* with SaPIbov1 *tst::tet*M PICI particles (*n* = 3, ***p < 0.001) and growth in media with tetracycline or *E. coli* infection with EcCICFT073 *c1504‐c1507::cat* PICI particles (*n* = 3, ***p < 0.001) in media with chloramphenicol. One‐way ANOVA with Tukey's multiple comparisons test was performed to compare mean differences within rows. Error bars represent the standard deviation of the mean. B) Detection of *S. aureus* using *µ*PADs with SaPIbov1 was used to test the exponential growth and colour development of transduced cells (with initial concentrations of ≈10^2^ and 10^1^ CFU mL^−1^). Controls were used with no bacteria added and no PICI added respectively. Images in each row represent the colour development after 4 and 10 h of the addition of PrestoBlue. C) Detection of *E. coli* 594 using *µ*PADs with EcCICFT073 was tested using ≈10^4^ and ≈10^2^ CFU mL^−1^. Results are representative images taken from three independent experiments where the growth of bacteria was enabled by the transduction of PICIs and detection through PrestoBlue development.

Having established the calibration curve and LODs, we tested the sensitivity of each PICI and the ability to detect between the two bacterial species in a mixed culture. For this, we mixed samples with *E. coli* 598 (10^2^ CFU mL^−1^) and *S. aureus* RN4220 (10^2^ CFU mL^−1^) and inoculated the paper devices containing EcCICFT073 or SaPIbov1 particles, respectively for each bacterial species. The detection on‐chip of *E. coli* was enabled by transduction of the EcCICFT073 *c1504‐c1507::cat* island and growth on media supplemented with 20 µg mL^−1^ chloramphenicol, while *S. aureus* cells were grown on devices supplemented with 3 µg mL^−1^ tetracycline by transduction of SaPIbov1 *tst*::*tet*M island. Images presented in **Figure** **5**A show color development and data obtention using PrestoBlue, ColorAnalyzer app and EasyRGB. The mean values gave an approximate 0.48 color intensity which corresponded to ≈300 CFU mL^−1^.

Having noticed that the development of color by PrestoBlue was exponentially increased by the growth of the bacteria over time, we decided to record the detection of cells at initial cell densities of ≈500 and ≈50 CFU mL^−1^ after 4 and 10 h of PrestoBlue addition. Note that the addition of PrestoBlue was performed after incubating devices at 37 °C overnight. Images of paper devices were taken from three independent experiments, demonstrating the continuous growth of bacteria and the development of color intensity, Figure 5B. To verify that development of PrestoBlue happens only when bacteria can grow; samples with no bacteria and PICI, samples with no PICI and bacteria, and samples with media were used as negative controls for this experiment, Figure 5B.

We determined the LOD of *E. coli* 594 and exponential growth on the paper devices with PrestoBlue added prior to incubation overnight. For this, we inoculated 5 × 10^4^ and ≈500 CFU mL^−1^ in the paper devices coupled with EcCICFT073 and antibiotic. Color development on the *µ*PADs was recorded with ColorAnalyzer and EasyRGB (Figure 5C). For devices inoculated with 5 × 10^4^ CFU mL^−1^, we obtained an average color intensity of 0.78 giving an estimate of 3.9 × 10^4^ CFU mL^−1^, while for those with 50 CFU mL^−1^ recorded a color intensity of 0.4 giving an estimate of 52 CFU mL^−1^. The sensitivity of the PICI‐based *µ*PADs could be enhanced with the addition of PrestoBlue after overnight incubation. However, adding PrestoBlue before incubating, yielded images that are more suitable for analysis, as the color development was evenly distributed throughout the device when comparing the color intensity of Figure 5B top row with Figure 5C bottom row.

### 2.5 Detection of *E. coli* by Melanin Production in PICI‐Based Devices

**Figure 6:**
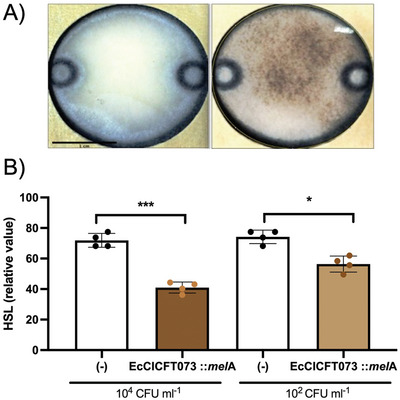
Melanin production on portable devices. A) Approximately 5 × 10^4^ CFU mL^−1^ of *E. coli* One Shot TOP10 were incubated in *µ*PADs containing LB supplemented with 20 µg mL^−1^ chloramphenicol, 15 µg mL^−1^ CuSO_4_, and 0.06 µg mL^−1^ *L*‐tyrosine and a high MOI of EcCICFT073 *c1498‐c1501*::*P_fhuD2_‐mel*A *c1504‐c1507*::*cat* lysate. After overnight incubation and development of melanin, images were acquired for analysis. These are representative of three independent experiments. Scale bar = 1 cm B) The relative value of HSL (Hue, Saturation, Lightness) were calculated. Graphs represent the mean of three independent experiments with four images acquired per sample on each experiment, where 100 is fully white and 0 is fully black. Statistical analysis was performed using one‐way ANOVA followed by Tukey's multiple comparison tests (*n* = 4 ± SD, ***p = 0.0003 for 10^4^ CFU, *p = 0.0107 for 10^2^ CFU). Error bars represent the standard deviation of the mean.

To avoid opening the paper devices and not relying on the color development of PrestoBlue, which requires specific reagents and storage, we used an engineered PICI capable of expressing *mel*A for the production of dark, melanin pigment, **Figure** **6**A. The paper‐based device was prepared with the required media for the development of the pigment as previously optimized by Gosset.^[^ ^48^, ^49^ ^]^ As a proof‐of‐concept, we detected a concentration of ≈5 × 10^4^ and ≈50 CFU mL^−1^ of *E. coli* on the device by analyzing the decrease in pixel intensity due to the production of melanin, Figure 6A,B. The LOD of our PICI‐melanin‐based device was estimated as ≈71.7% using the mean minus three standard deviations below the average control background (i.e., 73.10 − 3 × SD = 0.46). In relation to our previous set‐up using PrestoBlue, it was also determined experimentally using ≈50 CFU mL^−1^ as it gave an HSL value of 63%, Figure 6B.

## 3 Discussion

Phages provide a useful, specific vehicle that has been applied as bacterial detection technology.^[^ ^8^, ^9^, ^50^ ^]^ In this work, we demonstrate that phage‐like particles carrying MGEs as PICIs that parasitize phages can be integrated rapidly into their host cell and used as an important diagnostic platform. PICIs offer the advantage of infecting their host at different growth stages without causing negative effects such as lysis of the pathogen. Compared to plasmids, which are difficult to transfer/employ in point‐of‐care diagnostic strategies, they also offer greater stability as replication is not necessary. At the same time, they inherit the stability and specificity that their prophages have, making them suitable candidates for POC diagnostics. Here we developed PICIs into a sensitive, rapid, user‐friendly, portable test for infectious bacteria, which can be easily produced using printing technology and affordable materials.

We adopted a growth‐based assay platform previously fabricated by Funes‐Huacca^[^ ^8^ ^]^ and Deiss^[^ ^18^ ^]^ using portable self‐contained cultures for phage M13 and as antimicrobial susceptibility assays, which proved to be efficient at allowing bacteria to grow and be stored for several days. Our results establish that this type of portable device had similar LOD to assays performed in plate, Figure 3, suggesting that the performance is directed by the quantity of PICI and their ability to infect and transduce cells. We incorporated the use of a PrestoBlue viability reagent and a pre‐established medium to produce a melanin pigment as signal readouts. It is worth mentioning that optimizing concentrations of such reagent and medium compositions have the potential to improve signal development in future.

The use of PICIs can bypass issues observed with an active prophage, which severely affects the detection of viable cells. Samples treated with active phage particles had less than 1% of viable cells transduced and noticeably fewer viable cells than in the untreated control sample. The reduced number of viable cells could therefore have a negative effect on bacterial detection levels when trying to employ lysates with active helper phages, which stochastically could induce their lytic cycle.^[^ ^51^ ^]^ However, not all viable cells were detected with the transduced PICI DNA and antibiotic resistance gene, which may be due to either a subpopulation of viable cells not possessing the adequate conditions of infection (such as receptor availability) or expression of the reporter gene, and/or to mechanisms still unknown which could trigger cell death at high MOIs.

We also demonstrated that PICIs were more efficient at transducing antibiotic‐resistant markers than temperate phages and plasmids, reflecting the advantage of integrating into the chromosome and not causing lysis (Figure S2, Supporting Information).

The LOD of the paper microfluidic devices coupled with PICI transduction was ≈20 CFU mL^−1^ which is better than the recommended 10^4^ CFU mL^−1[^ ^52^ ^]^ for applications in food safety, for example. Additionally, we were able to use two different PICI species to detect both *E. coli* and *S. aureus* on‐chip as a duplex, Figure 5. Further experiments will be required to assess the LOD of these devices when attempting the detection of clinical isolates.

Our approach benefits from the fact that the detection readout is produced by the pathogen itself, and its preparation requires little equipment or training. Other methods that use phages or phage peptides do not have the ability to distinguish the pathogen directly^[^ ^53^, ^54^ ^]^ and others rely on phage reproduction to measure indirectly the presence of the pathogen^[^ ^55^, ^56^, ^57^, ^58^ ^]^ or exposed layouts unsuitable for field practices.^[^ ^59^ ^]^ To increase the sensitivity of our µPADs with PICIs, a pre‐enrichment stage could be used to concentrate bacteria from environmental samples and allowed them to enter the lag phase and exponentially grow above 20 CFU mL^−1^ to enable a successful PICI transduction. This approach was employed recently in a phage‐based portable assay to maximize phage infection and signal generation after 2 h of incubation.^[^ ^58^ ^]^

Future development of this approach could use engineered helper phages with diverse tail fibers to expand the delivery of the synthetic PICI DNA from different *E. coli* strains to other close‐related species (e.g., Salmonella spp., Pseudomonas spp., Vibrio spp., and Yersinia spp.). A similar approach was recently developed to deliver CRISPR‐Cas systems to modify the *E. coli* population in a mouse gut.^[^ ^60^ ^]^ In contrast to host expansion, other reporter genes in engineered PICIs could incorporate inducible promoters to trigger the signal as a response to the expression of a specific gene related to biofilm formation, virulence, or quorum‐sensing signals. This aspect could greatly improve sensitivity toward *S. aureus* isolates since PICIs have been reported to transduce intra‐ and inter‐specifically in Gram‐positive bacteria at a lower rate than their cognate species.^[^ ^61^ ^]^

Although we highlight that a PICI‐based detection system benefits the user for sequential recovery of the pathogen for further investigation, there are still some limitations such as the availability for the phage receptor to inject the PICI DNA,^[^ ^62^ ^]^ interference mechanisms from other MGEs^[^ ^63^, ^64^, ^65^, ^66^ ^]^ as well as the time required for bacterial growth to amplify the readout signal. In addition to general interference such as Restriction–Modification systems, a potential challenge that this application could face is the interference caused by the presence of a PICI already allocated on the integration site of the synthetic PICI used for detection.^[^ ^64^ ^]^ However, our experimental work has shown that PICIs can recombine their accessory modules more efficiently than prophages if selective pressure is presented, such as the addition of antibiotics.^[^ ^67^ ^]^ Multiple synthetic PICIs with different integrases and methylation patterns could be employed to bypass such issues. We are currently working on these concepts to optimize the detection system and improve the synthetic PICIs.

Our portable device satisfies the assured and reassured criteria^[^ ^68^ ^]^ either in part or completely, in terms of the diagnostic's capability of real‐time, ease of sample preparation, affordability, sensitivity, selectivity, user‐friendly, equipment‐free, and direct manipulation by the end‐user. Although the method currently needs longer incubation to amplify the signal than those based on DNA amplification by PCR,^[^ ^69^, ^70^ ^]^ it is a notable improvement over gold standard culture‐based approaches that require at least 12 h of incubation, without considering transportation time of samples, training of personnel, processes and analysis of results.^[^ ^2^, ^70^ ^]^

## 4 Experimental Section

### Bacteria Strains

The bacterial strains used are listed in Table S1 (Supporting Information). S*. aureus s*trains were grown at 37 °C on Tryptic Soy Broth (TSB), agar (TSA), or in TSB broth with shaking (180 rpm). *E. coli s*trains were grown at 37 °C on Lysogeny Broth (LB), agar, or in LB broth with shaking (180 rpm). Erythromycin (10 mg mL^−1^) chloramphenicol (20 mg mL^−1^) or tetracycline (3 mg mL^−1^; all Sigma‐Aldrich), were added when appropriate.

### PICI Induction

For *S. aureus*, an overnight culture in TSB was diluted to 1:50 in TSB and cultured in a shaking incubator at 37 °C and 120 rpm until 0.2–0.3 OD_540_. PICIs and phages were then induced by adding mitomycin C (1 mg mL^−1^) at a final concentration of 2 µg mL^−1^. For *E. coli* PICIs and phages, an overnight culture in LB media was diluted in 1:50 and cultured in a shaking incubator at 37 °C and 150 rpm until 0.15–0.17 OD_600_. PICIs and phages were then induced by adding mitomycin C (1 mg mL^−1^) at a final concentration of 1 µg mL^−1^. To induce and obtain higher titers of EcCICFT073, plasmid pJP2037 was employed, which carried alpA under the control of the P_BAD_ promoter. Donor strains were induced with 0.2% arabinose while adding mitomycin C to increase the packaging of EcCICFT073.

The cultures were incubated at 32 °C and 80 rpm for 3–4 h. Generally, lysis occurred at this time but to had a total lysed solution, the culture was left overnight at room temperature without shaking. To store lysates, the solution was filtered through a 0.2 µm filter (Minisart single‐use syringe filter unit, hydrophilic and non‐pyrogenic, Sartonium Stedim Biotech) and the phage stock was stored at 4 or −20 °C.

### PICI Transduction

For calculating the number of transductants in a lysate of PICIs and phages, an overnight culture of the acceptor strain was diluted 1:50 with fresh media and grown at 37 °C and 120 rpm until 1.4 OD_540_ or OD_600_. Once appropriately grown, 1 m CaCl_2_ was added to the culture (final concentration 4.4 mm). The PICI or phage lysates were established as serial dilutions using phage buffer from 10^−1^ to 10^−8^. In a sterile test tube, 1 mL of recipient cells were infected with 100 µL of the serial dilutions and incubated at 37 °C for 20 min. This incubation allowed the PICI or phage to infect the recipient strain.

After incubation, 3 mL of top agar (media + 3% agar) at 55 °C was added and immediately poured over the surface of a plate containing selective antibiotics and necessary nutrients. For *S. aureus*, TSA plates with antibiotic (10 µg mL^−1^ erythromycin or 3 µg mL^−1^ tetracycline) were used for selective culture of the successfully transduced bacteria with SaPIs or phages. For *E. coli*, LB plates with antibiotic (20 µg mL^−1^ chloramphenicol) were used for selective culture of the successfully transduced bacteria with EcCIs or phages. After the top agar had solidified (15–20 min), the plates were flipped and incubated at 37 °C for 24 h. Transduction titers of all lysates generated in this study are reported in Table S2 (Supporting Information).

### Design and Fabrication of Paper Microfluidic Devices

Wax patterns were designed using Inkscape (Free Open Source Software, GPL) or Microsoft PowerPoint 2016. A Xerox ColorQube 8570 Ink (Xerox Corporation, Connecticut, United States) printer was used to print the wax patterns as illustrated in Figure 1, for the media zone and the culture zone with two side‐zones for antibiotics. Once printed, the wax‐patterned Whatman paper was placed on a hot plate at 120 °C for 5 min.^[^ ^18^, ^71^ ^]^ Three layers of 12 cm 3 m scotch tape (48 mm wide) were cut and placed on top of each other as reinforcement. Using a Fiskars Medium Circle Lever Punch, a 1‐inch (2.54 cm) hole was made in the center of one of the halves of the 3‐layered tape. The edges of the tape layer were then folded over to create non‐sticky edges for opening and closing the device.

PDMS layers were fabricated with a mixture of silicone elastomer (Dow Corning, Amsterdam, Netherland) and silicone elastomer curing agent (Dow Corning, Amsterdam, Netherland) together in a ratio of 10:1 by pouring the mixture into a petri dish that was used as a mold. To achieve a 3 mm layer, 17 g of total silicone mixture was poured and then vacuum‐spun for 30 s at 1000 rpm. The petri dish molds were then placed into a vacuum flask, ensuring a tight closure, and vacuuming for 15 min. The vacuum was switched off and the mold was left in the vacuum flask retaining a constant air pressure for 15–30 min. The plate was then removed from the vacuum flask and left on top of the bench overnight to ensure a good polymerization of the PDMS. Once set, 3 × 3 cm squares were cut and used as the PDMS layer for the device. The PDMS layer was placed on top of the hole against the sticky side of the tape. The wax‐patterned culture zone was then placed against the PDMS window and pressed to seal it against the tape. Using the hole puncher, 3 circles of 1 inch were cut from the blotting paper (Amersham HybondGE). These blotting paper circles were implemented as the media reservoir for the device since evaporation moved toward the PDMS. These were then placed on the other half of the device with the wax‐patterned media zone on top, aligned with the culture zone so the media and culture zones could get in proper contact. Finally, the device was folded in half to align the zones. All portable devices were rinsed and sterilized before sealing the device for storage, appropriate media supplemented with antibiotics was added into the culture and media reservoirs, see Figure 2 for the procedure.

To comply with affordability from the WHO's ASSURED criteria,^[^ ^68^, ^72^ ^]^ a cost analysis of the components of the portable device was made. The portable devices were fabricated with low‐cost material including Scotch tape, blotting paper, wax patterned paper and PDMS. The price of fabrication per chip was calculated with a cost of goods of $0.1 (10 c, or £0.08) /device (this could be further decreased if a production line with bulk purchases were taken into account). Retail prices were based on suppliers used to order these materials in 2015–2016. Suppliers were Sigma‐Aldrich, VWR International and Amazon UK.

### Image Intensity Calculation

Images from portable devices were taken with an iPhone smartphone camera and processed with ColorAnalyzer Mobile App (Satoshi Nakamura, App Store) to measure the color intensity and light intensity. Routinely, the smartphone camera was placed ≈20 cm away from the samples. Within the ColorAnalyzer Mobile App, values for L (lightness), a* (green‐red scale) and b* (yellow‐blue scale) were recorded to then calculate the relative sRGB (standard Red Green Blue) value or the HSL (Hue, Saturation, Lightness) value using the color algorithm from EasyRGB website (http://www.easyrgb.com). The sRGB value used was relative to red color, where 0 equals green and 1 equals red, and the HSL value, where 0 was black and 100 was white. The color values obtained from EasyRGB were represented as squares on top of each plot point, illustrating the color development for each concentration of bacteria.

### Melanin Induction

To engineer the EcCICFT073 PICI carrying the *mel*A gene, PCR fragments were generated to assemble the PICI in a YAC for *S. cerevisiae* BY23849, which was then rebooted into *E. coli* 594 lysogen for *ϕ*80 ∆*cos*N.^[^ ^34^ ^]^ This gene was obtained from pTrcmelA^[^ ^48^, ^49^ ^]^ and subcloned into pCN51 with a constitutive promoter (*P_fhuD2_*). Transduction titers of the rebooted EcCICFT073 *c1498‐c1501::P_fhuD2_‐melA c1504‐c1507::cat* were tested and recorded on *E. coli* strains 594 and DC10B.

For detection of *E. coli* on paper microfluidic devices by melanin production, portable devices contained LB media supplemented with 20 µg mL^−1^ chloramphenicol, 15 µg mL^−1^ CuSO_4_, and 0.6 g L^−1^ *L*‐tyrosine, and 100 µL of *ϕ*80 ∆*cos*N EcCICFT073 *c1498‐c1501::P_fhuD2_‐melA c1504‐c1507::cat* lysate. A culture of *E. coli* One Shot TOP10 was serially diluted to ≈10^4^ CFU mL^−1^ and 100 µL was added to the culture zone of the *µ*PADs, closed and incubated at 37 °C overnight. On the next day, portable devices were observed under a Leica dissection microscope and images were acquired to examine the production of melanin and quantification of light intensity.

ImageJ software was used to create a surface plot representing the clusters of melanin dye representing colonies of *E. coli*, with images processed into single channel colour images for the calculation of the relative HSL values, using 6 images taken from 3 independent experiments, with ColorAnalyzer. The HSL values computed from the algorithm in the EasyRGB website were used to estimate the lightness affected by the dark spots of melanin on the portable device, where 100% represented fully white or light and 0% represented fully black or darkness.

### Data and Statistical Analysis

Raw data was organized on GraphPad Prism 6 (La Jolla). Phage and PICI titers were calculated as TFU mL^−1^ or CFU mL^−1^, respectively. Phage and PICI titer assays were performed at least in biological and technical triplicates. Expression assays on‐chip were performed at least in biological and technical triplicate. Results were shown as the mean with standard error of the mean (SEM). The data acquired from image analysis was obtained using ImageJ software^[^ ^73^ ^]^ (National Institutes of Health, Bethesda, MD, USA) and/or ColorAnalyzer Mobile App (Satoshi Nakamura, App Store). Sample size (*n*), means and, standard deviation (± SD) were represented in each figure. Assessment of statistically significant differences between groups was performed using a one‐way ANOVA followed by Tukey's multiple comparisons test on log10 transformed data. For each statistical analysis, all figures with the *** convention were indicated above the respective comparison and reported *p*‐values in the figure legends: (ns) not significant, (*) *p*‐value < 0.05, (**) *p*‐value < 0.01, (***) *p*‐value < 0.001, (****) *p* < 0.0001. GraphPad Prism 6 (La Jolla) was used for statistical analysis.

## Conflict of Interest

The authors declare no conflict of interest.

## Author Contributions

R.I.‐C., J.R.P., and J.M.C. conceptualized the study, R.I.‐C. carried out the experiments and drafted the manuscript. All authors analyzed the data and edited the manuscript.

## Supporting information

Supporting Information

Supplemental Table 1

## Data Availability

The data that support the findings of this study are available in the supplementary material of this article.
