# Supplementary material for: Phage‐Inducible Chromosomal Islands as a Diagnostic Platform to Capture and Detect Bacterial Pathogens
Source: Adv Sci (Weinh). 2023 Jun 26;10(24):2301643. doi: 10.1002/advs.202301643 (PMC10460865; doi:10.1002/advs.202301643)
Supplement: Supplementary file 1 — Supporting Information [file ADVS-10-2301643-s002.pdf]

ADVANCED SCIENCE
Open Access

Supporting Information

for *Adv. Sci.*, DOI 10.1002/advs.202301643

Phage-Inducible Chromosomal Islands as a Diagnostic Platform to Capture and Detect Bacterial Pathogens

Rodrigo Ibarra-Chávez, Julien Reboud, José R. Penadés and Jonathan M. Cooper**

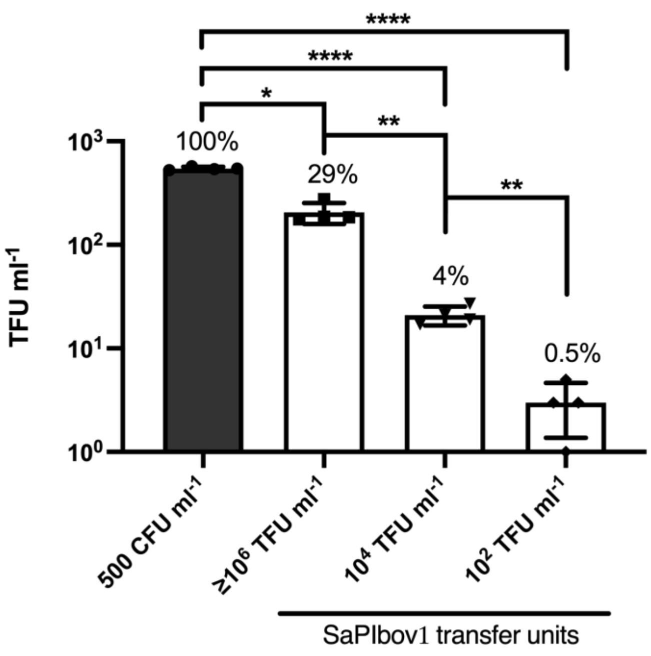

Figure S1. MOI effect on LOD. Graph shows the effect of MOI number on the LOD ~200 CFU mL^{-1}. The total of viable cells (black) is represented as 100%. Using different MOI numbers, the percentage of viable cells detected was calculated. SaPI lysate of 80α *Δter*S SaPIbov1 were used at 10^8 TFU mL^{-1} 10^6 TFU mL^{-1}, 10^4 TFU mL^{-1}, and 10^3 TFU mL^{-1}. Statistical analysis was performed using one-way ANOVA followed by Tukey's multiple comparisons test (n=4 ±SD, *p= 0.0118, **p= 0.0015, ****p= < 0.0001). Error bars represent the standard deviation of the mean.

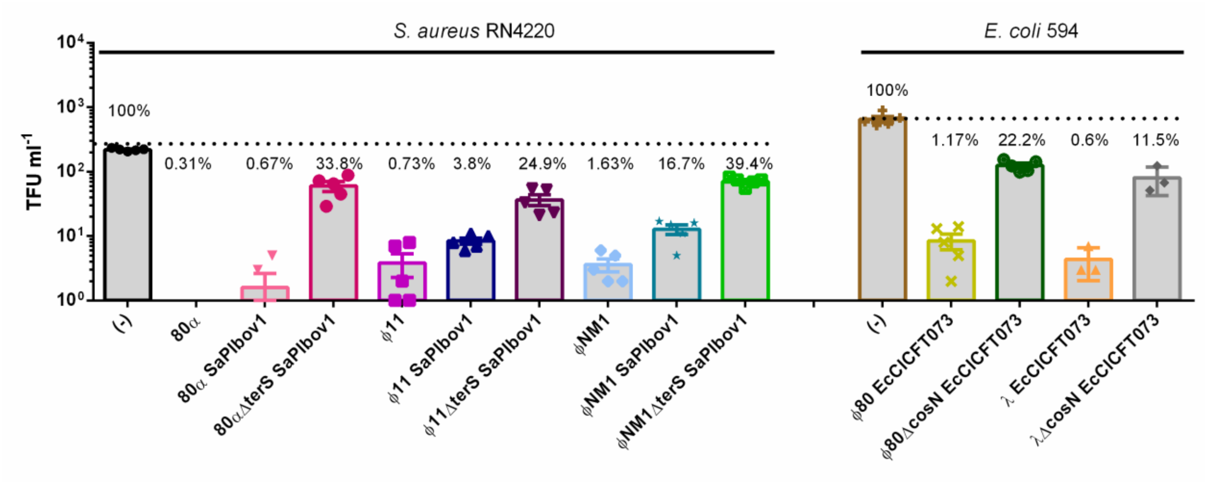

Figure S2. Phage and PICI sensitivity tests, percentages of detection. Graphs represent the ratio of transduced cells by phage or PICI infection to the total of viable cells used in the detection assays. PICI infection and integration was detected by tetracycline resistance in *S. aureus* and chloramphenicol resistance in *E. coli*, while phage integration was tracked by erythromycin resistance. Transduction of plasmid in *E. coli* was determined with chloramphenicol resistance. All infections occurred at an initial concentration of ~200 CFU mL^{-1} of RN4220 cells or ~500 CFU mL^{-1} of 594 cells with 100 μL of each lysate using a normalised titre of 10^6 TFU mL^{-1} for PICIs and prophages. Graphs show the means of six independent experiments with error bars representing the standard deviation (n=6 ±SD).

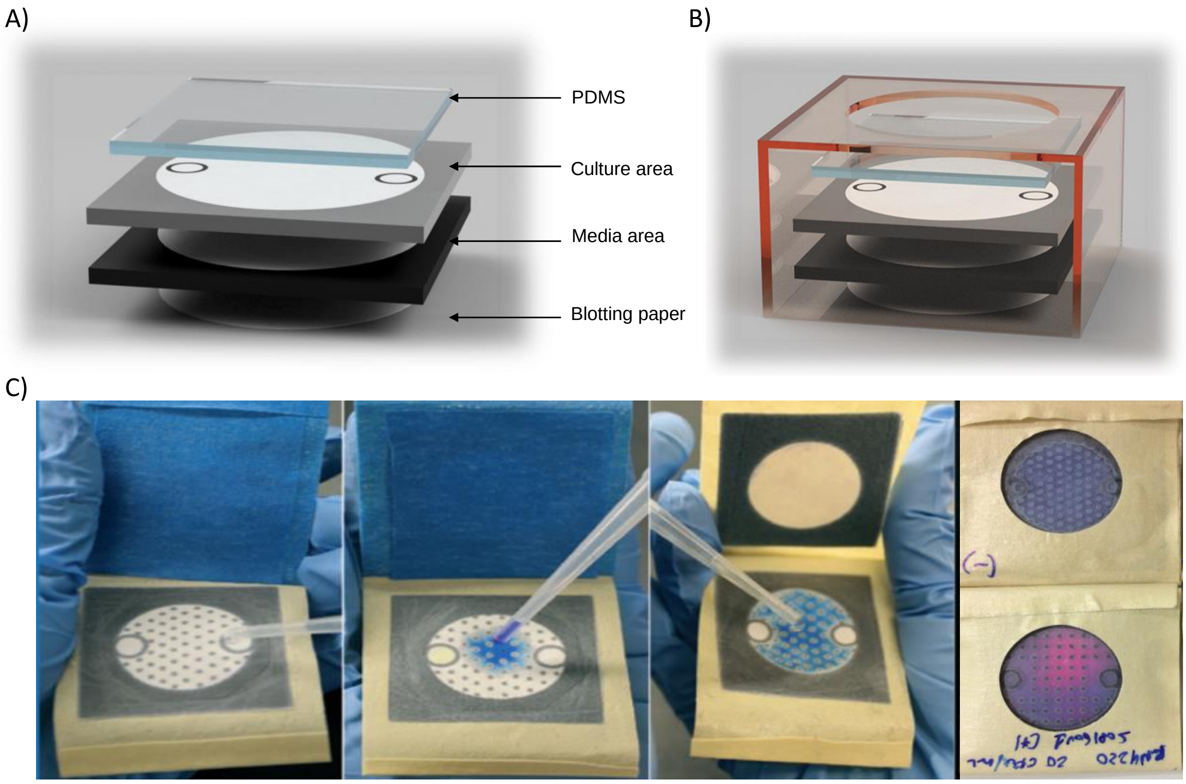

Figure S3. Layout of components for the assembly of paper microfluidic devices. A) Each component was fabricated separately and assembled onto adhesive tape. Blotting paper was first pasted onto the adhesive tape followed by the media area, whilst on the other side of the tape, the culture area and PDMS were assembled. **B)** From bottom to top; adhesive tape, blotting paper, wax-patterned media area, wax-patterned culture area, 5mm PDMS layer, and adhesive tape with 2.5 cm aperture punched away. **C)** Open layout, application, and signal development. Blue tape serves as a protective layer for application of selective media and PrestoBlue^{TM}. Then sample is allocated in culture area, closed, and incubated for growth and development of viability dye.

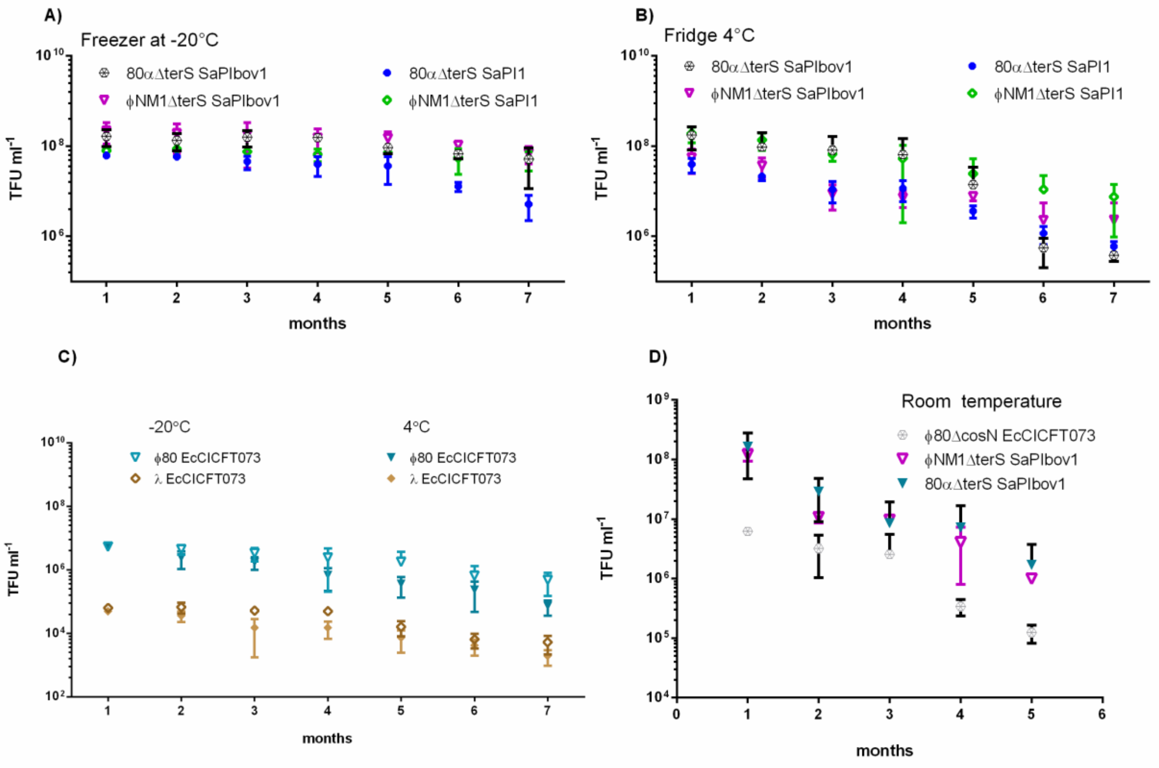

Figure S4. Long term storage effect of PICIs. Aliquots of **A)** SaPIbov1 and **B)** EcCICFT073 lysates were stored at -20°C and 4°C during a time course of 7 months. Transduction titres of lysates containing the indicated PICI lysates were tested each month by infecting either strain *S. aureus* RN4220 or *E. coli* 598 and plating on media with antibiotics. Time points represent the mean of three technical replicates of three independent experiments (n=3 ±SD). Error bars represent the standard deviation of the mean.

Table S1. Bacterial strains

Table S1. Strains used in this study

Specie	Strain	Description	Reference
S. aureus	**RN450**	NCTC8325 cured of φ11, φ12 and φ13	(Novick et al., 1967)
S. aureus	**RN451**	RN450 φ11	(Novick et al., 1967)
S. aureus	**RN4220**	RN450 restriction-defective	(Kreiswirth et al., 1983)
S. aureus	**RN10359**	RN450 80α	(Ubeda et al., 2007)
S. aureus	**JP6399**	RN4420 80α ::*erm*C	[74]
S. aureus	**JP6400**	RN4420 φ11 ::*erm*C	[74]
S. aureus	**JP12357**	RN4420 φNM1::*erm*C	This study
S. aureus	**JP3602**	RN10359 SaPI1 *tst::tet*M	(Tormo-Más et al., 2010)
S. aureus	**JP19819**	RN4220 80α SaPIbov1 *tst*::*tet*M	(Haag et al., 2021)
S. aureus	**JP13387**	RN4220 80α ∆*ter*S SaPIbov1 *tst:tet*M	(Haag et al., 2021)
S. aureus	**JP13388**	RN4220 80α ∆*ter*S SaPI1 *tst::tet*M	(Haag et al., 2021)
S. aureus	**JP21262**	RN10359 SaPIbov1 *sec*::*erm*C	(Haag et al., 2021)
S. aureus	**JP17410**	RN4420 80α ∆*ter*S SaPIbov1 *sec*::*erm*C	(Haag et al., 2021)
S. aureus	**JP1794**	RN451 φ11 SaPIbov1 *tst::tet*M	(Tormo-Más et al., 2008)
S. aureus	**JP3378**	RN451 φ11 ∆*ter*S SaPIbov1 *tst::tet*M	(Tormo-Más et al., 2008)
S. aureus	**JP18607**	RN4220 φNM1 SaPIbov1 *tst::tet*M	(Dearborn et al., 2012)
S. aureus	**JP18606**	RN4220 φNM1 SaPI1 *tst::tet*M	(Dearborn et al., 2012)
S. aureus	**JP17133**	RN4220 φNM1 ∆*ter*S SaPI1 *tst::tet*M	This study
S. aureus	**JP17134**	RN4220 φNM1 ∆*ter*S SaPIbov1 *tst::tet*M	This study
E. coli	**DC10B**	*mcrA* Δ(*mrr-hsdRMS-mcrBC*) φ80*lacZ*ΔM15 Δ*lacX*74 *recA*1 *araD*139 Δ(*ara-leu*)7697 *galU galK rpsL endA1 nupG* Δ*dcm*	(Schoenfelder et al., 2019)

E. coli	**594**	Laboratory strain derivative from K-12 ATCC10798	Laboratory strain CGSC#: 6132
E. coli	**C600**	ATCC23724, NCIB10222, F- *sup*E44 *lac*Y1 *thr*-1 *leu*B6 *mcr*A thi-1 *rfb*D1 *fhu*A21	Laboratory strain ATCC 23738
E. coli	**JP16617**	C600 **λ** EcCICFT073 *c1504-c1507*::*cat* pJP2037	(Fillol-Salom et al., 2018)
E. coli	**AF828**	594 φ80 EcCICFT073 *c1504-c1507*::*cat* pJP2037	(Fillol-Salom et al., 2018)
E. coli	**JP17091**	594 φ80 Δ*cos*N	(Ibarra-Chavez et al., 2020)
E. coli	**JP17094**	JP17091 EcCICFT073 *c1504-c1507*::*cat* pJP2037	(Ibarra-Chavez et al., 2020)
E. coli	**RIC242**	C600 **λ** Δ*cos*N	This study
E. coli	**RIC479**	JP19515 EcCICFT073 *c1504-c1507*::*cat* pJP2037	This study
E. coli	**RIC475**	JP17091 EcCICFT073 *c1498-c1501*::P_{fhuD2}-*mel*A *c1504-c1507*::*cat* pJP2037	This study

Table S2. Phage and PICI transfer rates

Phage	Phage-inducible chromosomal island	Phage titer [PFU mL^{-1}]	Transduction in plate [TFU mL-1]	Transduction in μPADS [TFU mL^{-1}]
80α Δ*ter*S	SaPIbov1	–	8.315x10^8	8.33 x10^7± 0.154
80α Δ*ter*S	SaPI1	–	7.99x10^8	6.014 x10^6± 0.27
Φ11 Δ*terS*	SaPIbov1	–	1.34x10^8	ND
ΦNM1 Δ*ter*S	SaPIbov1	–	1.75x10^9	7.84 x10^8± 0.242
ΦNM1 Δ*ter*S	SaPI1	–	9.9x10^8	ND
80α ::*erm*C	–	7.61x10^7	8.97x10^6	ND
Φ11 ::*erm*C		3.88x10^7	5.66 x10^6	ND
ΦNM1 ::*erm*C	–	1.23x10^8	3.66x10^7	ND
80α	SaPIbov1	8.35x10^8	1.19x10^7	ND
80α	SaPI1	6.31x10^7	7.99x10^6	ND
Φ11	SaPIbov1	1.2 x10^6	1.19x10^7	ND
ΦNM1	SaPIbov1	4.31x10^7	8.315x10^6	ND

ΦNM1	SaPI1	$7.33x10^{6}$	$7.99x10^{6}$	ND
λ	EcCICFT073	$7.34 x10^{7}$	$5.88x10^{6}$	$1.51 x10^{4} \pm 0.045$
80	EcCICFT073	$1.18 x10^{8}$	$1.46x10^{7}$	$7.301x10^{6} \pm 0.837$
80 Δ*cos*N	EcCICFT073	_	$6.34x10^{7}$	$4.76 x10^{6} \pm 0.52$
λ Δ*cos*N	EcCICFT073	_	$4.93x10^{6}$	$4.38 x10^{5} \pm 0.261$
80 Δ*cos*N	EcCICFT073 ::*mel*A	_	$5.21x10^{7}$	$6.46 x10^{6} \pm 0.345$

ND = no data
